# Effects of Polyethylene Terephthalate Microplastics on Anaerobic Mono-Digestion and Co-Digestion of Fecal Sludge from Septic Tank

**DOI:** 10.3390/molecules29194692

**Published:** 2024-10-03

**Authors:** Tingting Ma, Nana Liu, Yuxuan Li, Ziwang Ye, Zhengxian Chen, Shikun Cheng, Luiza C. Campos, Zifu Li

**Affiliations:** 1 Beijing Key Laboratory of Resource-Oriented Treatment of Industrial Pollutants, School of Energy and Environmental Engineering, University of Science and Technology Beijing, Xueyuan Road No. 30, Beijing 100083, China; matt10070124@163.com (T.M.); 13782039548@163.com (Z.Y.); chenzx0416@163.com (Z.C.); zifulee@aliyun.com (Z.L.); 2 Department of Civil, Environmental & Geomatic Engineering, University College London, London WC1E 6BT, UK; yuxuan-li.21@ucl.ac.uk (Y.L.); l.campos@ucl.ac.uk (L.C.C.)

**Keywords:** microplastics, anaerobic digestion, polyethylene terephthalate, fecal sludge, methane production, manure resource utilization

## Abstract

Anaerobic digestion (AD) is one of the most significant processes for treating fecal sludge. However, a substantial amount of microplastics (MPs) have been identified in septic tanks, and it remains unclear whether they impact the resource treatment of feces. To investigate this, polyethylene terephthalate (PET) was used as an indicator of MPs to study their effect on the anaerobic digestion of fecal sludge (FS). Two digestion systems were developed: FS mono-digestion and FS co-digestion with anaerobic granular sludge. The results indicated that the effects of PET varied between the two systems. PET inhibited volatile fatty acid synthesis in both systems, but the inhibition period differed. During mono-digestion, PET slightly increased gas and methane production, in contrast to the co-digestion system, where PET reduced methane production by 75.18%. Furthermore, in the mono-digestion system, PET increased soluble chemical oxygen demand and ammonia nitrogen concentrations while blocking phosphorus release, whereas the co-digestion system showed the opposite effects. Ultimately, the choice of digestion method is crucial for the resource utilization of septic tank sludge, and the impact of MPs on AD cannot be ignored.

## 1. Introduction

Plastic products, integral to our daily lives and various production activities, play a pivotal role in modern society. However, their mass production and resistance to biodegradation have introduced significant environmental risks, exacerbated by the rapid increase in plastic manufacturing [1]. Studies indicate that the globe currently generates more than 350 million tons of plastic per year, and it is predicted that around 25 million tons of plastic waste will be generated by 2050, of this waste, 94% is expected to be discharged into the environment in various ways [2,3]. Microplastics (MPs), defined as synthetic polymers less than 5 mm in size, have been proven to affect critical physiological functions in animals, including energy metabolism, respiration, growth, reproduction, and survival [4,5,6]. Various plastic items and microplastic particles from human excreta accumulate in septic via drains, becoming massive pools of plastic waste [7]. Liu et al. identified 36 types of MPs in septic, with concentrations ranging from 1489 to 4816 MPs/g of dry sludge, primarily in the form of fibers, beads, granules, and fragments [8]. This finding implies that septic tanks serve as both a source and a sink for microplastics. For example, fecal sludge (FS) is the main route of microplastic excretion in the human body, accounting for 94% of the total daily microplastic excretion, and most of these microplastics enter septic tanks [9]. The projected amount of MPs entering Chinese fields through FS is 7.8 × 10^3^–5.6 × 10^4^ tons/year [7], indicating that septic systems should be taken into account.

The septic system is acknowledged as an effective and long-term option for collecting domestic sewage in rural (and certain peri-urban and urban) regions. This system not only removes suspended organic matter from domestic wastewater primarily through sedimentation and anaerobic digestion, but also serves as a primary transitional domestic treatment structure [10,11]. After initial settling, the effluent’s upper layer retains a high concentration of organic matter, with total chemical oxygen demand, five-day biochemical oxygen demand, nitrogen, and pathogenic indicator microorganisms several times higher than those of domestic sewage. Meanwhile, the bottom layer accumulates sludge, which must be regularly removed and treated [12,13]. According to statistics, 44% of global wastewater is discharged into the environment without safe treatment, and polluted drinking water and sanitation cause 1.4 million fatalities per year [14,15,16]. Therefore, it is extremely important to manage FS safely to prevent disease dissemination and environmental pollution. Anaerobic digestion (AD) is considered as an effective approach not only for resource recovery in the form of bioenergy and bio-fertilizer, but also for contaminant stabilization and pathogen reduction, which has high prospects for application [17,18,19,20].

AD typically involves four sequential but coexisting stages: hydrolysis, acidogenesis, acetogenesis, and methanogenesis. These stages require a variety of enzymes and microorganisms working together to convert complex organic matter into methane and CO_2_ [21]. The process is influenced by several parameters, including temperature, pH, carbon/nitrogen ratio, and enzyme activity [22,23,24]. MPs have been demonstrated to be highly biotoxic, and they can form composite pollutants when combined with other pollutants, thereby altering the structure and function of microbial communities [25,26]. Research has revealed that microplastics significantly impact hydrogen, acid, and methane generation during anaerobic digestion. For example, Zhang et al. discovered that polyethylene (PE) could inhibit hydrogen production, with the inhibition effect being proportional to concentration and particle size [27]. Conversely, Zheng et al. found that PE could promote acid production at the start of digestion, but still exhibited an inhibitory effect under long-term stress [28]. Some other studies suggest that microplastics’ effect on methane production varies based on concentration and other factors [23,29,30,31]. For example, 10 particles/g TS of polyvinyl chloride (PVC) increased methane production by 5.9 ± 0.1%, but higher levels of PVC reduced production [31]. Polyethylene terephthalate (PET), the most common MP species found in septic tanks [8], has been shown to induce a shift in the microbial community in sludge in an unfavorable direction for hydrolysis-acidification, thereby inhibiting methane synthesis [29,32,33].

However, in previous studies, the feedstock used to examine the effect of MPs on AD was derived from municipal sludge (waste-activated sludge from sewage treatment plants). No study has examined the impact of adding MPs to the AD of FS yet. In this research, PET is utilized as the indicator microplastic and developed two digestion systems: FS mono-digestion and FS co-digestion with anaerobic granular sludge (AGS). To demonstrate how anaerobic digestion affects pollutants in FS, the concentration of chemical oxygen demand (SCOD), ammonia nitrogen (NH_3_-N), and total phosphorus (TP) were tracked before and after digestion under both scenarios. The dynamics of volatile fatty acid (VFA), which are significant intermediate products of anaerobic digestion, along with the cumulative methane and CO_2_ yield, were investigated to determine the effect of PET on the efficacy of FS replenishment.

## 2. Results and Discussion

### 2.1. Anaerobic Mono-Digestion

#### 2.1.1. Changes in Pollutant Concentrations

**Figure 1:**
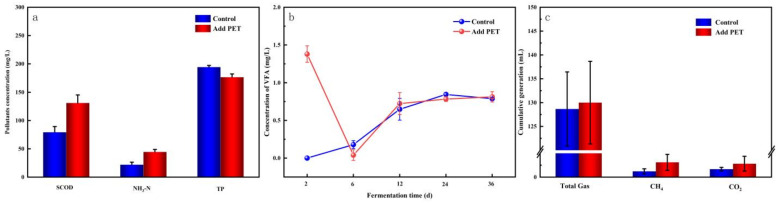
Indicators of mono-digestion system: (**a**) pollutants concentration changes; (**b**) volatile fatty acid (VFA) concentration; (**c**) gas, methane, and CO_2_ generation.

To evaluate the influence of anaerobic digestion on pollutants in septic tank sludge, the concentrations of SCOD, NH_3_-N, and TP before and after digestion were measured. The results indicated that the anaerobic process increased the concentration of each pollutant; however, the degree of change varied significantly among the pollutants. Therefore, the concentration at the end of digestion was subtracted from the initial concentration, and the differences in concentration changes before and after digestion are shown in Figure 1a.

During digestion, particulate organic matter in sludge decomposes and hydrolyzes, resulting in the solubilization of organic compounds into the liquid phase, as evidenced by the increased SCOD levels [34]. The SCOD concentration in the mono-system with PET addition increased more from 79.33 ± 10.02 mg/L to 131.00 ± 14.17 mg/L with a relative increase of 65.13% as compared to the control group. This increased SCOD indicates floc disruption, consistent with the findings of Wei et al. [31]. When waste-activated sludge was subjected to varying concentrations of PVC microplastics, the unknown fraction of SCOD rose with increasing PVC concentration. This phenomenon is speculated to result from the solubilization of extracellular polymers by lipids and nucleic acids released following microbial cell lysis, in addition to bisphenol A leaching from PVC. The fact that the addition of PET causes the concentration of SCOD to be significantly higher than that of the blank group could be explained by the fact that PET leaches dibutyl phthalate (DBP), diisobutyl phthalate (DIBP), and other chemicals to some extent [29].

Human feces and urine are great sources of nitrogen and phosphorus pollution. It has been estimated that the daily mean concentration of nitrogen in urine ranges between 8.8 and 9.2 g N/L for nitrogen, and 0.74 and 2 g P/L for phosphorus [35,36]. Urine contributes to the majority of the nutrients found in wastewater, with 79% of nitrogen and 47% of phosphorus (even higher when phosphate detergents are outlawed) [37]. The ranges of nitrogen and phosphorus in feces were found to be 1.1–18% and 0.39–4.93% of total sulfur, respectively [38]. Total nitrogen (TN) refers to the total amount of inorganic and organic nitrogen in various forms. Despite the potential for nitrate conversion to nitrogen, it remains in the system during anaerobic digestion. Only a small amount of nitrogen is converted into cells because anaerobic microbial cells proliferate is minimal. The majority of biodegradable organic nitrogen undergoes hydrolysis to form NH_3_-N nitrogen during anaerobic digestion explanation, primarily in the form of free NH_3_-N and ammonium ion NH_4_^+^-N [39].

Figure 1a shows that adding PET to septic tank sludge increased the concentration of NH_3_-N from 22.00 ± 4.35 mg/L to 44.33 ± 4.51 mg/L, representing 100% as compared to the control group during solo fermentation. The primary reason for elevated NH_3_-N concentrations in septic is microbial ammonification [40]. The primary source of NH_3_-N comes from the anaerobic process of hydrolysis of solid organic matter, or the breakdown of amino acids. The significant rise in NH_3_-N concentration can be explained by PET’s capacity to stimulate organic matter hydrolysis, as indicated by the increase in SCOD concentration under the same digestion conditions. Thus, microbial ammonification is the key reason for elevated NH_3_-N concentrations in septic tank sludge.

Phosphorus in sludge is transferred from the solid to the liquid phase by phosphorus-containing bacteria, which release phosphorus, shatter cells, and cleave extracellular polymers. According to relevant studies, the available phosphorus in urine and feces produced in urban environments was about 880,000 metric tons in 2011 and will increase to more than 1.5 million metric tons by 2050 as the population grows [41]. There exists significant potential for the recovery of phosphorus from human excreta. Figure 1a shows that the addition of PET reduced total phosphorus content from 194.4 ± 2.9 mg/L in the control group to 176.6 ± 5.7 mg/L in a mono-system, indicating a 9% inhibition rate of phosphorus release. This result can be explained by PET’s ability to suppress the growth of phosphorus-aggregating bacteria as well as the activity of phosphorus-releasing enzymes, resulting in the blocking of phosphorus transfer from the solid to the liquid phase. Phosphorus in sludge is mostly composed of organic phosphorus and inorganic phosphorus. Inorganic phosphorus can react with iron/aluminum ions to generate non-apatite inorganic compounds, as well as with calcium ions to form apatite inorganic compounds [42]. There has been little research on the release and recovery of phosphorus from PET during anaerobic digestion, which may be one of the future directions of the research.

In summary, PET-MPs had varying effects on feedstocks during FS mono-digestion. They promoted the release of SCOD and NH_3_-N, while the increase in elemental phosphorus concentration was lower in the presence of PET compared to the control, indicating a minor inhibition of phosphorus release.

#### 2.1.2. Changes in VFA Concentrations

Methane production is primarily driven by the consumption of VFA produced by methanogens, with only a minimal contribution from CO_2_ and H_2_. Nevertheless, the production of CO_2_ and H_2_ requires the intermediary step of forming VFA from macromolecular organic matter; hence, VFA is a significant intermediate product in the anaerobic digestion process [43]. The variation in VFAs between control and mono-digestion is depicted in Figure 1b. At the onset of the digestion process, the experimental group with PET pellets exhibited a VFA concentration of 1.38 mg/L, whereas the control group had a VFA concentration of 0 mg/L, indicating significantly higher levels in the experimental group. The VFA concentration in the experimental group rapidly decreased to zero on the sixth day of digestion. Meanwhile, the experimental group’s VFA concentration was lower than that of the control group, which had been increasing since the second day of digestion. Subsequently, VFA levels in both the control and experimental groups progressively rose, and the difference in VFA concentration between them progressively shrunk until it was roughly equivalent to 0.8 mg/L. The analysis suggests that: PET promotes the production of acids during the anaerobic digestion of FS, particularly the production of VFAs during the pre-digestion phase (12 days prior to the pre-digestion period and the post-digestion period).

#### 2.1.3. Methane Production

Methane accounts for 50–80% of biogas, which also comprises CO_2_ (20–40%), nitrogen, hydrogen, oxygen, and a minor quantity of hydrogen sulfide. Figure 1c illustrates the influence of PET on methane and CO_2_ generation during anaerobic mono-digestion of FS. The anaerobic mono-digestion of FS can produce a certain amount of gases, including methane, CO_2_, and other components, as depicted in the figure. However, due to poor digestion performance, less methane, CO_2_, and total gases were produced than in the previous study [18,44]. PET has a negligible impact as well, offering a modest increase. Specifically, the experimental group produced 130.00 ± 8.66 mL of gas following the addition of PET pellets, representing a 1.02% increase compared to 128.67 ± 7.77 mL in the control group without PET. According to Figure 1c, less than 5% of the total gas in both groups was produced as CO_2_ and methane, indicating that the total gas production performance of single septic tank sludge is relatively low. This implies that the total gas generation performance of a single septic tank sludge is subpar. PET exhibited a modest beneficial effect on methane production, with the experimental group demonstrating a higher proportion of methane compared to the control group. Overall, the experiment’s findings demonstrated that PET could improve the production of all gases, including CO_2_ and methane, in a mono-digestion system. Additionally, the ratio of CO_2_ to methane was higher in the experimental group than in the control group, indicating that PET had a favorable effect on methane production.

### 2.2. Anaerobic Co-Digestion

#### 2.2.1. Changes in Pollutant Concentrations

**Figure 2:**
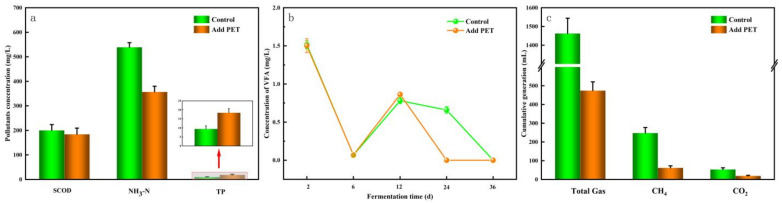
Indicators of co-digestion system: (**a**) pollutants concentration changes; (**b**) VFA concentration; (**c**) gas, methane, and CO_2_ generation.

The difference in pollutant concentrations before and after the co-digestion of septic tank sludge and AGS outcomes is opposite to those observed in mono-digestion. Figure 2a shows that the increase in SCOD concentration in the experimental group after adding PET was smaller than in the control group. The control group’s SCOD concentration increased by 200 ± 23.52 mg/L, while the PET-treated group’s increase was 184 ± 25.24 mg/L, a little drop from the control. This is consistent with Wang et al.’s experimental findings [29], which demonstrated that SCOD concentrations in experimental groups with 30 μm and 250 μm PET were reduced by 7.37–8.62% and 2.46–6.17%. The SCOD concentration in the experimental group remained consistently lower than in the control group throughout the digestion process, indicating that PET can restrict the breakdown of organic matter during anaerobic digestion. Our results further indicated that under the conditions containing abundant anaerobic microorganisms, although the concentrations of SCOD in the system were all increasing, PET may slightly inhibit cell lysis and organic matter release, resulting in a lower increase in SCOD concentration in the experimental group compared to the control group.

NH_3_-N is produced during the biodegradation of proteins or other nitrogenous compounds. During co-digestion, the NH_3_-N concentration increased by 538.67 ± 67 mg/L in the control group, while it decreased by 32.0% to 356.50 ± 23.73 mg/L in the experimental group with PET addition. This variation is consistent with the changes in SCOD. Appropriate ammonia levels are essential for maintaining system stability during anaerobic digestion and providing buffering capacity for active microbial processes [45]. However, excessive NH_3_-N levels can lead to systemic toxicity and hinder anaerobic digestion [46].

In the mono-system, PET inhibited phosphorus release, whereas, in the co-digestion system, PET promoted phosphorus release, with concentrations ranging from 9.4 ± 1.7 mg/L to 18.4 ± 2.3 mg/L. This suggests that PET may facilitate the release of phosphorus and the leaching of organic materials containing phosphorus. In a study by Liu et al., microplastics had no significant effect on the activity of polyphosphate-accumulating organisms (PAOs) [47]. Similarly, He et al. reported that PS microplastics in the range of 0–300 μm did not affect the activity of PAOs [48]. Our study did, however, demonstrate that PET microplastics affected the concentration of TP in both mono-digestion and co-digestion systems. As a result, future research efforts should focus on the inter-transformation and mechanisms of phosphorus in various forms.

#### 2.2.2. Changes in VFA Concentrations

Figure 2b illustrates the fluctuation in VFA concentration during a co-digestion using AGS and FS. PET had no discernible effect on VFA content during the early phases of the combined anaerobic digestion of FS and AGS. In the later stages of digestion, the VFA concentration in the experimental group with PET added decreased to zero earlier than in the control group, indicating that PET particles had a more pronounced effect during the final stages of co-digestion. This observation contrasts with the inhibition period seen in mono-digestion. This could be because the presence of PET in the reaction system hindered the activity of acid-producing bacteria, resulting in a decrease in VFA synthesis in the later stages of digestion. Consequently, the VFA concentration in the system was insufficient to support the methanogenic phase, causing the concentration to drop to zero earlier.

In a study by Zheng et al., the adverse effects of synergistic toxicity of high concentrations of PS particles with external pollutants via sodium dodecyl sulfate (SDS), high levels of reactive oxygen species (ROS), and aged microplastics outweighed the positive effects of solubilization, resulting in the inactivation and inhibition of microbial and VFA production [49]. The AGS introduced a significant amount of anaerobic flora into the system, and despite PET slightly inhibiting the anaerobic process, the sludge digestion performance remained adequate. This is demonstrated by the fact that the VFA concentration in the experimental group initially reduced to zero in the late stages of digestion, indicating that the activity of methanogenic bacteria was greater than that of acid-producing bacteria, leading to the rapid conversion of VFA in the system.

#### 2.2.3. Methane Production

Figure 2c depicts a comparison of methane, CO_2_, and total gas production accumulation after 36 days of co-digestion operation. The control group without PET pellets produced 247.61 ± 29.57 mL of methane and 53.45 ± 8.87 mL of CO_2_ during digestion. PET particles increased methane and CO_2_ production by 61.45 ± 11.07 mL and 18.70 ± 3.18 mL, respectively, with an inhibition rate of 75.18% and 65.01%. This suggests that PET had a significant inhibitory effect on methane and CO_2_ production from anaerobic digestion in the mixed system, stronger than that found by other researchers in their papers. Furthermore, PET greatly reduces the overall quantity of gas generated. According to Wang et al.’s study on the effect of PET on anaerobic digestion of food waste, PET with an abundance of 2.70 mg/g TS (about 0.170 particles/g TS) had the highest inhibitory performance of 21.6% on cumulative methane production [29]. This inhibitory effect was shown to be connected with PET particle size, with larger particles having a less significant inhibitory effect, a difference that might be attributed to the digestion sludge’s properties as well as the particle size of the microplastics. Since both steps are interconnected, it is commonly acknowledged that the methanogenic step is more vulnerable to unfavorable environmental factors and that inhibiting the acidogenic step can eventually worsen methanogenesis [50].

By researching studies linked to microplastics and anaerobic digestion, three major ways were discovered on microplastics that alter sludge resources.

(1) Enzymes activity: It is well-known that the anaerobic digestion process necessitates the involvement of several enzymes. Proteases and α-glucosidases break down proteins and polysaccharides into amino acids and monosaccharides. BK converts amino acids to SCFAs, while AK converts acetyl coenzyme A to acetic acid. Acetic acid is then methylated using coenzyme 420 [23]. Microplastics can influence anaerobic reactions by changing the activity of important enzymes [49,51]. The active site distribution of enzymes in sludge may also have an effect on the anaerobic process, as the protection of extracellular polymers (EPS) limits the chance of enzyme–substrate contact and hence affects enzyme effectiveness [52,53].

(2) Microplastic leachate: DBP, the major leachate in PET microplastics, was added to the California Proposition 65 (1986) list of probable teratogens in 2006, and it is thought to be an endocrine disruptor with high cytotoxicity [54]. In tests involving the generation of hydrogen, Wei et al. [32] substituted DBP for PET and discovered that the inhibitory characteristics of DBP were equivalent to those of PET, with the inhibitory impact becoming stronger at increasing concentrations. Wang et al. discovered that DBP reduced the abundance of key critical hydrolyzing bacteria (*Bacteroides vadin HA 17*) and acidifying bacteria (*Clostridium* and *Sphaerochaeta*) [29]. A decrease in important acid-producing bacteria (like *Leviloptera* sp.) and methanogenic bacteria (like *Methanosaeta* sp.) was discovered in another experiment on AGS chronically exposed to PET, which helps to explain how PET prevents the synthesis of methane [33].

(3) ROS: The vast surface area of microplastics enables a high percentage of reactive groups to be present on their surfaces. These groups can react with molecular oxygen to form reactive ROS through catalytic processes and free radicals [55]. Submicromolar oxygen is still present in the medium even in anaerobic settings, where it can interact with the many active sites on the surface of microplastics to produce ROS through disproportionation and Fenton reactions [56]. Organisms protect themselves from moderate oxidative stress using their natural antioxidant systems, but higher levels of oxidative stress can cause a pro-inflammatory response by activating redox-sensitive signaling pathways, causing cell death [57]. PET microplastics at 150 MP/L and 300 MP/L increased ROS by 12.5% and 17.3%, respectively, in Zhang et al.’s study [33]. These results were visually confirmed by technical tests conducted on living and dead cells.

All of the explanations mentioned above can be employed in this study to explain the influence of PET on methanogenic outcomes, but this research concentrates on the effect of PET on the anaerobic digestion of septic tank sewage. Changes in acid generation, methane production, and certain significant pollutants were identified in both mono- and co-digestion situations. Based on the aforementioned analysis of Figure 2c, it can be concluded that PET inhibited methane generation in the co-digestion by 75.18%, and the fraction of methane in total gases produced was also lowered.

### 2.3. Environmental Impact of the Two Digestion Systems

**Figure 3:**
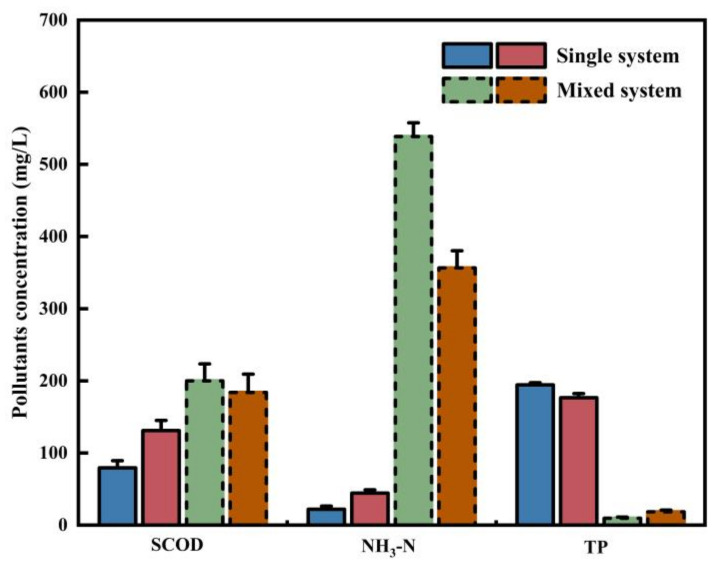
Comparison of contaminant concentrations in different digestion systems.

The concentration growth of contaminants in the two digestion scenarios is compared, and the findings are shown in Figure 3. SCOD, an indicator of dissolved organic matter in sludge, increased more in the co-digestion system than in the mono-digestion, at rates of 153.2% and 40.5%, respectively. This finding suggests that mixed fermentation of septic tank sludge with AGS can significantly increase cell lysis and organic matter release. However, the inclusion of PET alters the relative rate of change. The concentration changes of ammonia nitrogen followed the same trend as that of SCOD, with both showing larger concentration increases in the co-digestion system than in the mono-system. The co-digestion system increased ammonia nitrogen concentrations by 538.67 ± 18.9 mg/L and 356.5 ± 23.7 mg/L, much greater than the mono-system’s 22 ± 4.36 mg/L and 44.33 ± 4.51 mg/L, respectively. This data completely demonstrates the effect of the digestion system on the concentration of ammonia nitrogen. Although compound digestion can boost digestion efficiency, it also raises the environmental risks of septic tank sludge. TP concentration variations were consistently in the opposite direction of SCOD and ammonia levels. In the co-digestion of AGS and septic tank sludge, the increase in the concentration of total phosphorus was as low as 9.67 ± 1.7 mg/L and 18.43 ± 2.3 mg/L, respectively. The increase in the mono-system was 20 and 10 times higher than that in the co-digestion system. This finding proves that co-digestion can significantly minimize phosphorus release and subsequent treatment pressure, but it has a detrimental effect on phosphorus recovery. Overall, the digestion scenario significantly influenced the changes in pollutant concentrations during the anaerobic digestion of septic tank sludge. When compared to mono-digestion, co-digestion resulted in much larger increases in SCOD and NH_3_-N concentrations. The concentration of NH_3_-N in the co-digestion system was 8.1–24.5 times higher than in the mono-system, which could pose serious impacts on aquatic ecosystems if discharged into the environment without treatment [58,59].

**Figure 4:**
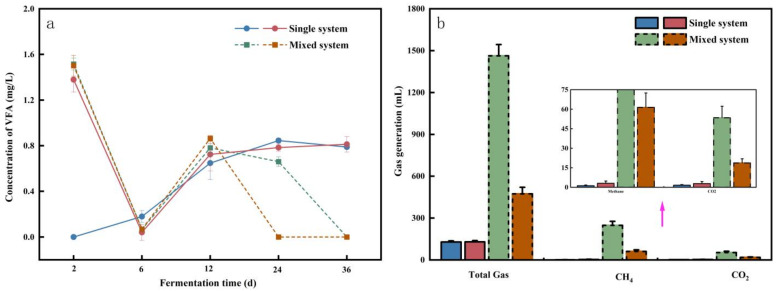
Comparison of acid and cumulative gas/methane production in different digestion systems: (**a**) Comparison of VFA concentrations between the two digestive systems; (**b**) Comparison of gas generation between the two digestive systems.

Similarly, the acid and gas generation rates of the two digestion systems are compared in Figure 4. Figure 4a depicts the change in VFA concentration in the system with digestion time. The experimental groups with PET added in the co-digestion system and the mono-system had almost identical concentration changes in the pre-digestion period, but there was a significant distinction after the 12th day of digestion. At the end of digestion, the co-digestion system’s VFA concentration was down to 0 mg/L, whereas the mono-system’s VFA concentration was 0.8 mg/L. This could be due to the addition of AGS, which introduces more methanogenic bacteria into the AGS as well as the nutrients required to carry out the anaerobic reaction, in which VFA is used in huge numbers to generate methane [60,61]. In contrast, the reaction within a mono-system is slower and does not quickly use VFA for methane generation.

Figure 4b represents the results of the gas, methane, and CO_2_ production comparisons in the two systems, and it is obvious that the co-digestion system has a substantially larger ability to produce total gas and methane than the mono-digestion without the addition of PET. The co-digestion system produced 11 times more total gas, 205 times more methane, and 32 times more CO_2_ than the mono-system, with a significant increase in the proportion of methane and CO_2_ in the total gas after adding the AGS.

The composition of AGS is complex, and after the digestion process of producing acid, the generation of methane precursors is also complex and involves a greater number of archaea. Due to these unique characteristics and the coexistence of fungi and archaea in the sludge, the archaea are easily influenced by environmental factors, and the microbial population of the co-digestion will be significantly higher than that of a mono-digestion [62]. The currently described methanogenic bacteria are classified into three trophic classes: (1) 62 deoxidizing bacteria, which oxidize H_2_ and CO_2_ to produce methane; (2) 20 methanogens, which use methyl compounds such as methanol, methenamine, and dimethyl sulfide; and (3) nine acetic acid-producing methanogens, which produce methane from acetic acid [63]. Although AGS can significantly increase gas and methane production, the presence of PET still has an inhibiting effect, as indicated in Section 2.2.3. The preceding discussion demonstrates that while the choice of digestion system is critical for the resource efficiency of septic tank sludge, the effect of PET cannot be overlooked.

## 3. Materials and Methods

### 3.1. Sludge and PET Microplastics

In this study, septic tank sludge was collected from a non-sewer public toilet in Beijing that is emptied once every seven days, and AGS was obtained from WUNIDIAN. AGS and septic tank sludge raw materials were gravity-thickened and kept at 4 °C before usage. The number of microplastics in septic tank sludge can reach 2803 (1489–4816) particles/g dry sludge, and they are diverse; a total of 36 different types of microplastics were detected. Table 1 displays septic tank sludge and AGS’s fundamental characteristics.

The PET-MPs for experiments, in the form of white transparent granules, were purchased from Shanghai Plastics (Shanghai, China). According to the manufacturer, the size of PET-MPs was 1 mm. Confirmation of microplastic size using a microscope (SZX2-ILLB, Olympus). Ten PET-MP particles were picked from the samples, and the morphology of these MPs was studied and photographed using a microscope (brand, model), and the details are visible in Supplementary Materials.

### 3.2. Anaerobic Digestion

**Figure 5:**
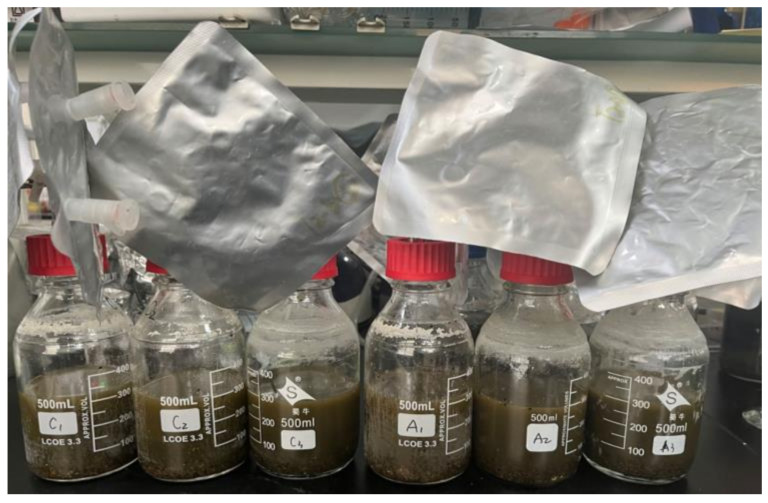
AD reaction bottles.

Mono-digestion system: 500 mL serum bottles filled with 100 mL septic tank sludge and 200 mL ultrapure water; 36 PET microplastic particles (at a concentration of 3 particles/g-TS) were added to the experimental group, and the blank group had no PET. Co-digestion: 500 mL serum vials filled with 100 mL septic tank sludge, 232 mL AGS, and 100 mL ultrapure water with a vs. ratio of 2.0 ± 0.1, the experimental and blank group is the same as mono-digestion. The actual anaerobic digestion reaction bottles are shown in Figure 5, and three parallel experiments were set up for each group, for a total of four groups and 12 digestion reaction bottles, which were performed simultaneously. The digestion was done in a water bath (37 ± 1 °C) and bottles were shaken manually every day to ensure even sludge distribution. All tests lasted approximately 36 days until the gas produced was minimal. The gas in the gas bags was removed during the experiment per the amount of sludge gas produced to analyze its components. Simultaneous sludge samples were collected to track any variations in the concentrations of TP, NH_3_-N, SCOD, and VFA.

### 3.3. Analytical Methods

Determination of TS, and vs. according to standard methods. Before analyzing water quality indicators such as SCOD, TN, NH_3_-N, and TP, the sampled sludge was first centrifuged (VELOCITY 18RPRO, Shanghai), filtered using an aqueous filtration membrane with a pore size of 0.45 μm, and evaluated using the standard methods of HACH reagent (HACH, Shanghai). VFAs were analyzed using high-performance liquid chromatography (Agilent, American) and samples were filtered through a 0.22 μm filter membrane and detailed in the Supplementary Materials. As shown in Figure 5, a gas bag is used to collect the gas produced by anaerobic digestion. When sampling and analysis are required, the gas is drawn out of the bag by a syringe to measure the volume (the valves between the bag and the reaction bottle are closed before drawing the gas to prevent air from entering), and the gas components are analyzed using a gas analyzer (GEM5000, England).

### 3.4. Quilty Control

All tests were repeated three times, with synchronized digestion, and the results were averaged and reported as mean values. Throughout the experiment, care was taken to avoid introducing other microplastics that could potentially alter the results.

## 4. Conclusions

In this study, two distinct digestion scenarios were simulated to investigate the impact of PET on the anaerobic digestion of FS. In the mono-digestion system, PET promotes the release of two pollutants, SCOD and NH_3_-N nitrogen, while inhibiting the release of phosphorus. PET could also inhibit the production of VFAs in the early stage of digestion but had no effect in the later stage. It promoted the production of methane in gas production. In contrast, the co-digestion system exhibited opposite results: phosphorus was released, whereas SCOD and ammonia concentrations were inhibited. Acid production was primarily inhibited in the later stages of digestion, and methane and CO_2_ gas production were significantly reduced. Ultimately, the choice of digestion method is critical for the resource recovery of FS, and the impact of PET, which is present in large quantities in fecal sludge, on digestion effectiveness should not be underestimated.

This study focuses for the first time on the influence of microplastics on FS and investigates the effect of anaerobic fermentation on pollutant concentrations in the sludge system. It also revealed that PET has a substantial effect on the anaerobic fermentation of septic tank sludge for replenishment, based on the findings of conventional methane production trials, which is directly related to the fermentation method used. Furthermore, we believe that the follow-up to this work can be pursued in the following two directions:

(1) Look into how PET influences the anaerobic fermentation of fecal sludge through deeper processes (microbial populations, important enzyme activity, reactive oxygen species, additive leaching, etc.).
(2) To examine the impacts of several types and particle sizes of MPs on anaerobic digestion of FS in septic tank sludge, which includes a large number and variety of microplastics, and to see if they have synergistic effects.

## Figures and Tables

**Table 1:** Fundamental characteristics of fecal sludge (FS) and anaerobic granular sludge (AGS).

	VS/(%)	TS/(%)	SCOD/(mg·L^−1^)	NH_3_-N/(mg·L^−1^)	TP/(mg·L^−1^)
FS	31.96	6.07	1251.00	386.00	77.20
AGS	27.55	6.80	225.00	171.00	6.20

## Data Availability

The data presented in this study are available on request from the corresponding author.

## Supplementary Materials

The following supporting information can be downloaded at: https://www.mdpi.com/article/10.3390/molecules29194692/s1, Figure S1: (a) PET-MP pellets used for the experiment; (b) electron microscopy of PET-MP particles.
