# Supplementary material for: Effects of Polyethylene Terephthalate Microplastics on Anaerobic Mono-Digestion and Co-Digestion of Fecal Sludge from Septic Tank
Source: Molecules. 2024 Oct 3;29(19):4692. doi: 10.3390/molecules29194692 (PMC11478245; doi:10.3390/molecules29194692)
Supplement: Supplementary file 1 [file molecules-29-04692-s001.zip › molecules-3184424-supplementary.pdf]

Supporting Information

Effects of Polyethylene Terephthalate Microplastics on Anaerobic Mono-Digestion and Co-Digestion of Fecal Sludge from Septic Tank

Tingting Ma [1], Nana Liu [1,*], Yuxuan Li [2], Ziwang Ye [1], Zhengxian Chen [1], Shikun Cheng [1,*], Luiza C. Campos [2] and Zifu Li [1]

[1] Beijing Key Laboratory of Resource-Oriented Treatment of Industrial Pollutants, School of Energy and Environmental Engineering, University of Science and Technology Beijing, Xueyuan Road No.30, Beijing 100083, China

[2] Department of Civil, Environmental & Geomatic Engineering, University College London, London WC1E 6BT, UK

* Correspondence: nanaliu@xs.ustb.edu.cn (N.L.); chengshikun@ustb.edu.cn (S.C.)

Support information includes the following:

Number of pages: 3

Number of tables: 0

Number of figures: 1

1. Electron micrographs of PET-MPs:

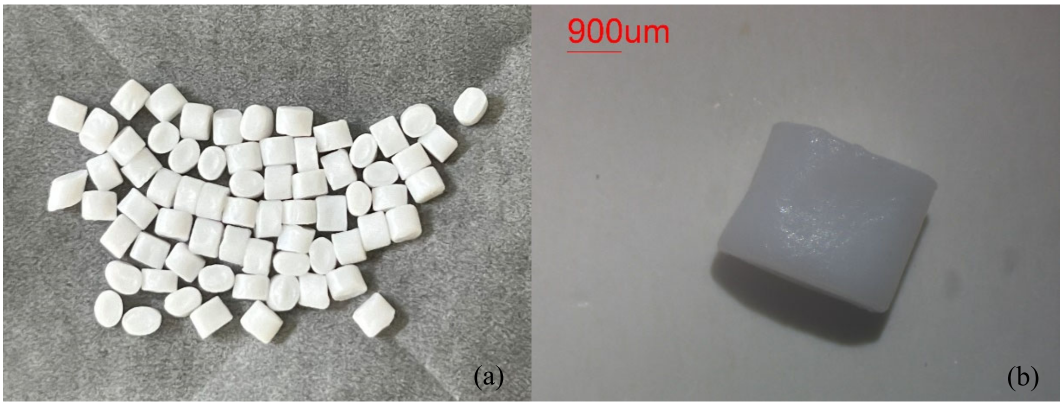

Figure S1. (**a**) PET-MP pellets used for the experiment; (**b**) electron microscopy of PET-MP particles

2. Analysis of TS/VS:

Bake the crucible in the oven at 105 ℃ until constant weight (2 h) and record the mass M_0, weigh about 3 g of the sample to be tested into the crucible and record the mass M_1, put the crucible and the sample into the oven at 105 ℃ for 24 h and record the mass M_2.

$$TS = \frac{M_2 - M_0}{M_1 - M_0}$$

The crucible which was scorched to constant weight (about 2 h at 550 ℃) was weighed G_0, about 3 g of the dried sample was weighed with a balance, the exact weight G_1 was recorded and put into the crucible, the crucible was scorched in a muffle furnace at 550 ℃ for 2 hours, it was removed and cooled down to room temperature and then weighed G_2.

$$TS = \frac{G_1 - G_2}{G_1 - G_0}$$

3. Gas components analysis:

A biogas analyzer (QED Environmental Systems, GEM5000) was used to analyze the gas components produced by anaerobic fermentation. The gas inside the gas bag was withdrawn with a 200/500 ml syringe and quickly connected to the inlet pipe of the biogas analyzer, and the gas fraction test was carried out according to the method of use of the biogas analyzer.

4. Quantitative analysis of VFAs:

Liquid chromatography was used to analyze VFAs in fermentation sludge quantitatively. The supernatant of sludge fermentation was passed through a 0.22 μm filter membrane before sampling. The composition of VFAs was determined according to the time of peaks, and the

concentration of each component was determined according to the peak area. The parameters of liquid chromatography were as follows:

Column: C18 column (InertSustain C18 5 μm 4.6×150 mm);

Injection volume: 10 μL;

Column temperature: 30 ℃;

Detection: UV λ = 210nm;

Mobile phase: pure methanol + potassium dihydrogen phosphate buffer 10 mmol/L (15:85 v/v, pH = 2.5);

Flow rate: 1 mL/min;

Phosphate buffer configuration: 1.56g sodium dihydrogen phosphate dihydrate plus ultrapure water to 1L, pH adjusted to 2.5 with phosphoric acid.

5. SCOD/NH_3-N/TP:

They are measured using HACH reagents, measurement methods available from the HACH producer.
